# Reconstructive Options after Oncological Rhinectomy: State of the Art

**DOI:** 10.3390/healthcare11121785

**Published:** 2023-06-16

**Authors:** Andrea Migliorelli, Rossella Sgarzani, Giovanni Cammaroto, Andrea De Vito, Manlio Gessaroli, Marianna Manuelli, Andrea Ciorba, Chiara Bianchini, Stefano Pelucchi, Giuseppe Meccariello

**Affiliations:** 1 ENT & Audiology Unit, Department of Neurosciences, University Hospital of Ferrara, 44100 Ferrara, Italy; 2 DIMES Department, Bologna University, 40100 Bologna, Italy; 3 Plastic Surgery, M. Bufalini Hospital, AUSL Romagna, 47521 Cesena, Italy; 4 ENT Department, Morgagni Pierantoni Hospital, AUSL Romagna, 47121 Forliì, Italy; 5 Head-Neck and Oral Surgery Unit, Department of Head-Neck Surgery, Otolaryngology, Santa Maria delle Croci Hospital, AUSL Romagna, 48121 Ravenna, Italy; 6 Maxillo-Facial Unit, M. Bufalini Hospital, AUSL Romagna, 47521 Cesena, Italy

**Keywords:** rhinectomy, nasal reconstruction, oncological rhinectomy

## Abstract

Background: The nose is a central component of the face, and it is fundamental to an individual’s recognition and attractiveness. The aim of this study is to present a review of the last twenty years literature on reconstructive techniques after oncological rhinectomy. Methods: Literature searches were conducted in the databases PubMed, Scopus, Medline and Google Scholar. “Preferred Reporting Items for Systematic Reviews and Meta-analysis (PRISMA)” for scoping review was followed. Results: Seventeen articles regarding total rhinectomy reconstruction were finally identified in the English literature, with a total of 447 cases. The prostheses were the reconstructive choice in 213 (47.7%) patients, followed by local flaps in 172 (38.5%) and free flaps in 62 (13.8%). The forehead flap (FF) and the radial forearm free flap (RFFF) are the most frequently used flaps. Conclusions: This study shows that both prosthetic and surgical reconstruction are very suitable solutions in terms of surgical and aesthetic outcomes for the patient.

## 1. Introduction

The nose is a central component of the face, and it is fundamental to an individual’s recognition and attractiveness [1,2,3,4]. It is well documented in the literature how a deformity or total absence of the nose has a strong negative impact on the psychosocial well-being of the individual [2,3,4]. Nowadays, the most frequent indication for total rhinectomy is nasal malignancies. About 2000 cases of nasal tumors are diagnosed in the USA per year and account for 3% of all head and neck cancers [1,5]. The most frequent histologies are basal cell carcinoma and squamous cell carcinoma [5,6,7,8]. Nasal reconstruction is one of the oldest techniques in plastic surgery. The earliest written texts on nasal reconstruction techniques come from India between 1000 and 600 BC, in which some surgical fundamentals used even today are described. In Europe, on the other hand, the first treatise on plastic surgery was written in 1500 by Gaspare Tagliacozzi of Bologna, who in his “De Curtorum Chirurgia” described in detail some surgical techniques for nasal reconstruction [9]. In the 20th century, the development of modern biocompatible materials has led to the increasing use of prostheses for nasal reconstruction [9]. Despite centuries of history, nasal reconstruction still represents a great challenge even for the most expert surgeons. To date, there are two main reconstructive options that we can propose to a patient after total rhinectomy: surgical reconstruction and prosthetic rehabilitation [1,3,10]. The aim of this study is to present a literature review on reconstructive techniques after oncological rhinectomy, attempting to investigate and better understand the role, limitations, aesthetic and functional results of the various treatment options.

## 2. Methods

**Figure 1:**
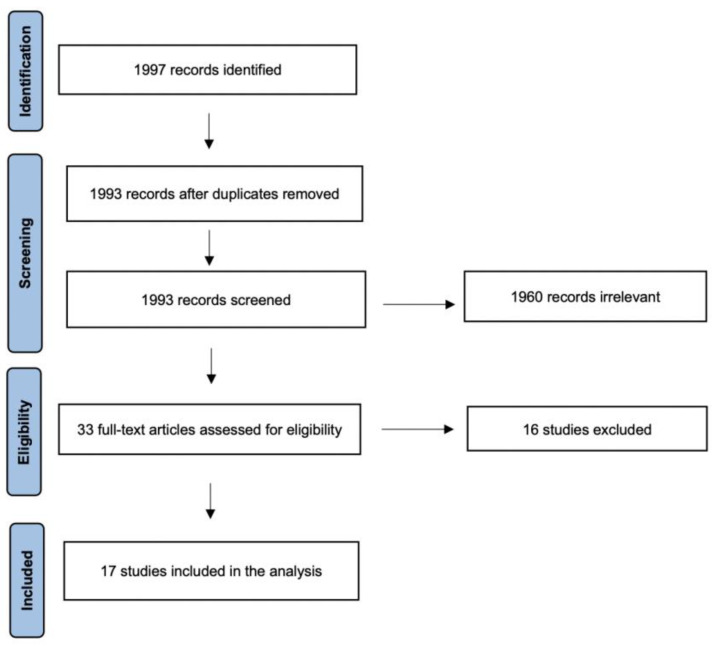
PRISMA flow-chart of the scoping review.

A detailed review of the English literature on nasal reconstitution following subtotal or total rhinectomy for oncological reasons was performed using PubMed, Medline, Scopus and Google Scholar databases. The literature search was carried out in accordance with the guidelines mentioned in “Preferred Reporting Items for Systematic Reviews and Meta-analysis (PRISMA)” for scoping review [11] (Figure 1). Two searches were performed using the keywords “Rhinectomy” and “Total Nasal Reconstruction”. The search yielded a total of 1997 relevant articles on this topic. We included only English-language articles, published from January 2002 to August 2022, with abstracts and containing a series of 5 or more patients. Furthermore, we only considered patients who underwent nasal reconstruction for oncological reasons. The number of cases, sex, age, histology, type of reconstruction, number of procedures, follow-up period, complication and aesthetic outcome were collected and compared in the present review. Articles without abstract or where data were missing have been excluded from the study. Papers where the type of nasal reconstruction and/or the etiology leading to the rhinectomy was not specified were also excluded. After title and abstract screening, 33 papers were left. At the end of the full-text review, 17 studies were included for a total number of 447 cases [1,2,3,4,6,7,8,10,12,13,14,15,16,17,18,19,20].

## 3. Results and Discussion

In this scoping review, seventeen articles regarding total rhinectomy reconstruction matched our inclusion criteria, for a total of 447 cases. The result of our review is summarized in Table 1. Of the 447 patients included in the review, we have information about the gender on 390, 215 (55.1%) of whom were males and 175 (44.9%) females. At the time of surgery, the mean age range was 44.5–71.6 years. Basal cell carcinoma (177/394) and squamous cell carcinoma (174/394) were the histologies which most frequently led to demolition surgery of the nose; out of 53 patients, we do not have the histological features.The other histologies reported are malignant melanoma, Merckel cell carcinoma, desmoplastic squamous cell carcinoma, skin appendage carcinoma, epidermoid carcinoma, malignant fibrous histiocytoma or recurrent carcinoma. Our review showed that the reconstruction with prosthesis was performed in six studies and surgical reconstruction in 8, while three studies used both techniques. The prostheses were the reconstructive choice in 213 (47.7%) patients, followed by local flaps in 172 (38.5%) and free flaps in 62 (13.8%). The most frequently used local flap is the forehead flap (FF), which may also be bipedicles or associated with a septal pivot flap. On the other hand, the radial forearm free flap (RFFF) is the free flap with the highest casuistry (50/62), followed by the anterolateral thigh free flap (ALTFF) (12/62). The reconstructive surgical stage may be performed either in the same session as the demolition stage or may be delayed by months. This decision is based on the surgeon’s experience and the stage of the tumor. The number of procedures performed for reconstruction varies according to the technique chosen. The follow-up time range was 12 months–5 years. Out of 213 patients treated with prostheses, 14 prostheses failures, 4 periimplantitis, 2 screw losses and 1 infection were observed. Bulky flap surgery (12/172), flap failure (8/172), nasal flap obstruction (7/172), venous congestion (3/172), dehiscence (1/172) and cartilage infection (1/172) are the complications observed in patients reconstructed with local flaps.

Following RFFF, eight cases of venostasis required surgery, and there were six cases of partial necrosis and four of necrosis. No complications were described in the 12 patients who underwent ALTFF. In seven of the studies examined, the aesthetic result was analyzed using different questionnaires, with good results achieved in all cases [1,3,4,7,10,13,14].

### 3.1. Clinical and Treatment Features of Nasal Malignancies

Malignancies of the nose are rare neoplasms, accounting for less than 10% of head and neck cancers, with an annual incidence in the United States of 0.5–1.0 per 100,000 people [21]. SCC is the most frequent histology in this location.

In our review, there is a slight predominance of males (215 males and 175 females), with an average age between the fifth and seventh decade of life. The most frequent oncological etiology is basal cell carcinoma (177) followed by squamous cell carcinoma (174), sporadic cases of melanoma or other rare tumors are also described (such as Merckel cell carcinoma, desmoplastic squamous cell carcinoma, skin appendage carcinoma, epidermoid carcinoma, malignant fibrous histiocytoma or recurrent carcinoma).

Smoking is the main risk factor; other demonstrated risk factors are exposure to wood dust, nickel and possibly chemicals used in leather processing [21,22,23]. Recently, many studies have focused on the role of the human papillomavirus (HPV) in the genesis of these tumors. HPV has been found in 25% of patients with nasosinusal SCC, with a better prognosis than in HPV-negative tumors, as has also been found in other head and neck sites [21,22,23]. To date, however, the role of HPV in nasal cavity SCC remains unclear. Concerning staging, three main classifications are currently used in the literature: the Wang classification, the 8th edition of the AJCC staging system for nasal cavity and ethmoid sinus tumors and the AJCC staging system for non-melanoma skin tumors of the head and neck region [24,25]. The nasal cavity is by far the most common site for neoplasms of epithelial origin arising in this region. Since SCCs of the nasal cavities are rare, according to the current TNM classification system, nasal cavity carcinomas are classified in the same classification as ethmoid carcinomas [25]. This has led to discordant results in the literature, considering that nasal cavity tumors have a significantly better survival rate than sinus tumors. Furthermore, it is important to emphasize that even early stage (T2) tumors may show bone invasion and thus lead to extensive treatment with important functional and psychosocial impact. The most frequent presenting symptoms are epistaxis, nasal obstruction and facial pain [21,22,23]. Being highly non-specific symptoms, they are often misinterpreted, resulting in an important diagnostic delay. Indeed, most patients present with advanced tumors. Due to the diagnostic delay, local aggressiveness and rapid growth propensity, the management of these tumors is often challenging. Achieving local tumor control is the main challenge in SCC of the nasal cavities and the preferred therapy is still controversial.

Nowadays, surgery is considered the gold standard treatment for nasal carcinomas with better oncological results than radiotherapy [1]. Advanced nasal tumors often require total or subtotal rhinectomy, which may be associated with neck dissection. If performed with wide resection margins, this surgery provides good control of the disease [26]. The outcome of this procedure, which is necessary in order to obtain oncological radicality, creates facial disfigurement with a consequent negative aesthetic and psychosocial impact [27].

Over the centuries, numerous surgeons have focused on which nasal reconstructive possibilities could be offered to patients undergoing rhinectomy [9]. There are currently two rehabilitation options found in the literature: prosthetic rehabilitation and surgical reconstruction. 

To the best of our knowledge, with 447 patients analyzed, this is one of the most comprehensive reviews reported in the literature on nasal reconstruction after oncological rhinectomy. Regarding reconstruction methods, the scientific community is more or less equally divided between surgery (234 cases) and prosthetics (213 cases). The decision of which of the two techniques to choose depends on several factors, such as the age of the patient, the size of the defect, the past medical and surgical history, the patient’s prognosis and the preferences of the patient and the surgeon [28].

### 3.2. Surgical Techniques

Surgery is one of the widely used and described options for nasal reconstruction. Several techniques of surgical reconstruction are described in the literature, all with better aesthetic and functional results. Based on the available literature, it is difficult to assess which reconstructive method is preferable, as each surgeon makes adaptations to standard techniques based on his or her own experience and the resources available at the center where he or she works [1,3,4,7,10,12,14]. However, all the articles unanimously agree that reconstruction of a total nasal defect should include the reconstitution of each of the three layers of nasal tissue: the inner mucosal layer, the support layer and the outer skin [1,3,4,7,10,12,14,29,30,31,32].

Surgical reconstruction of the inner nasal lining is challenging and can be performed mainly with three alternatives: skin graft, mucosal graft and local or free flaps. The use of a skin graft often results in stenosis and breathing difficulties due to scarring. Buccal or turbinate mucosal flaps often have excellent results even if the available tissue is limited [2,30].

Once the reconstruction of the inner layer is finished, it is required to give a new shape to the nose, trying to achieve the best possible aesthetic result. Therefore, the surgeon must now focus on reconstructing the structural support layer [30]. Cartilage and bone from the septum (if not infiltrated by the tumor), ribs and auricular cartilage can be used as structural grafts. The decision concerning which graft should be used depends on the individual case and the surgeon’s preference. Moreover, in the literature, promising results have been found from preliminary studies involving the reconstruction of the structural support layer by custom-made titanium plate created by computer-aided design (CAD) and computer-aided manufacturing (CAM) technologies [33].

For reconstruction of the outer layer, either a skin graft or the portion of outer skin donated from the flap can be used [30].

Therefore, surgical reconstruction is often performed using local flaps or free flaps [1,3,4,7,10,12,14]. Moreover, in RFFF, there is also the advantage of being able to use a radial bone component for reconstruction of the structural layer.

Thus, a reconstructive surgeon should be able to choose between the different techniques in order to design a tailored approach for the patient [29]. 

#### 3.2.1. Local Flaps Technique

Our review shows that surgical reconstruction is performed in most cases using a local FF, and only in a small percentage of cases, a free flap is used [29]. Paddack et al. in 2012 [16] published their experience of 107 patients treated with local flaps (FF or nasolabial flap), with a failure rate of 5.6%, observing that the tendency for failure was higher in smoking patients. 

Due to the quality of its color and texture, the skin of the forehead has been recognized as the best donor site for nose reconstruction [13]. In the traditional FF technique, the procedure is performed in two steps [13,16].

In the study by Paddack et al. [16], FF is performed by a traditional two-stage technique, using the Doppler probe to determine the length of the pedicle and relying on the supratrochlear artery. The flap is initially raised in the subcutaneous or subgaleal plane, depending on the thickness required, until the pedicle portion of the flap is reached. At this point, the flap is processed in the subgaleal plane, including the frontalis muscle with the pedicle. After elevation, the flap is carefully cut and inserted with minimal tension into the recipient site. FF donor sites are managed by primary closure. Either autologous rib cartilage, allogeneic rib cartilage or synthetic materials can be used to support the flap. Three to four weeks after the graft, the second stage is performed, in which the flap is thinned again when the pedicle is divided up to the junction of the proximal and distal halves. The pedicle is now amputated, and the closure is performed in order to improve symmetry and allow for a more aesthetically pleasing result. If further sculpting is necessary, this is usually performed as a third stage.

Ribuffo et al. [13] compared the traditional two-stage procedure with the three-stage surgery. In both cases, a Doppler probe is used to analyze the quality of the vessels. The first stage is the same in both techniques and consists of lifting a full-thickness flap of the forehead without thinning it (except for the columellar area). In the 2-stage technique, 3 weeks after the first step, the pedicle of the flap is divided without further thinning or with minimal thinning. The two-stage technique, although faster, almost inevitably required a new procedure in the following months and years to achieve a good aesthetic result. In contrast, the three-stage technique involves a second stage three weeks after the first procedure in which the skin and subcutaneous fat are lifted and thinned, with the exception of the columellar area. In addition, the underlying muscle and cartilage are shaped to create a good rigid matrix on which the thin skin is overlaid. The third stage involves the pedicle section three weeks after the second stage (6 weeks after the first operation). The authors conclude stating that the three-stage method for FF nasal reconstruction allows for a better final three-dimensional structure, as close as possible to the real nose, than the classic procedure. Although this technique takes extra time, thus extending the total time from surgery to the final result, it is compensated by a better aesthetic result and the minimal need for further revisions. Furthermore, the three-stage technique seems to be more suitable for defects that include bone and/or cartilage tissue, as the flap has a better blood supply than the traditional technique. Therefore, this technique should be the surgical gold standard for smoking patients.

#### 3.2.2. Free Flaps Technique

Regarding free flaps, Krakowczyk et al., in 2020 [7] published a case series of 48 patients treated with the RFFF, which is also the most frequently used (50 cases) in our review. Nasal reconstruction using the RFFF requires the preparation of the vascular pedicle, based on the radial vessels, together with three skin islands. In addition, it is also possible to obtain approximately 1/3 of the circumference of the radial bone to reconstruct the dorsum and columella. This allows a three-dimensional reconstruction of the nose. Autologous costal cartilage can be harvested to support the flap. The outer surface of the skin islands is temporarily covered with skin grafts. Anastomoses are made with the facial or temporal vessels. The second stage involves external nasal reconstruction and is performed on average 8 weeks after the first stage. This step involves the creation of an FF to cover the created structure. After a further 4 weeks, the third stage is carried out in which the pedicle of the FF is dissected. Occasionally, to achieve an optimal aesthetic effect, the patient requires further surgical corrections to achieve improvements in nasal shape and symmetry. The RFFF has the advantage of being thin, flexible, hairless and easy to elevate, although donor site morbidity can lead to undesirable adverse effects [7].

The use of ALTFF is also described in the literature with good aesthetic and functional results, although with only 12 cases reported it needs further studies to confirm its validity [2,14,20]. In all patients receiving ALTFF described by Livaoğlu et al. [2], a preoperative Doppler ultrasound for the study of the perforations was performed. The cutaneous island was delineated according to the size of the defect. The anastomoses were all performed end to end with the facial vessels. The ALTFF has the advantage that the procedure can be performed by two teams working simultaneously, thus reducing operating time. Furthermore, due to its pliability, pedicle length and thickness, it is a widely used free flap for reconstructing head and neck defects.

Anastomosis of the free flaps is usually performed with the facial vessels [7]. 

#### 3.2.3. Advantages and Disadvantages of the Surgical Option

The advantages of the surgical technique consist mainly in a better match of skin color and texture. It also provides a permanent solution without the need for maintenance procedures that prostheses require [23]. On the other hand, disadvantages include an increased risk of surgical complications and complications associated with the graft site, such as bulky flap surgery, flap failure, nasal flap obstruction, venous congestion, dehiscence and cartilage infection [7,13,16,17,19]. Furthermore, it should be considered that reconstruction often requires more surgical procedures than the application of the prosthesis. Therefore, patients with multiple comorbidities may not be candidates for this type of reconstructive approach, indeed many articles select patients with ASA lower than 3 [13,19]. Finally, it should be emphasized that there is no unanimity in the literature on the timing of flap reconstruction, with some authors performing it concurrently with the demolition time by performing an intra-operative frozen-section examination of the margins, while others recommend postponing it for about a year if radiotherapy is required or if total local control of the disease is uncertain [2,7,14,15]. In the latter case, the prosthesis can be a bridge solution between rhinectomy and surgical reconstruction [19]. 

### 3.3. Prosthetic Aid

In our review, 47.7% of patients were treated by a prosthesis application. The main indications for the prosthesis are patients who do not wish to undergo further reconstructive surgery, elderly patients with multiple morbidities, high perioperative risk under general anesthesia and doubts about complete resection of the tumor [12]. 

Moreover, the use of prostheses does not delay post-operative radiotherapy, which can generally be started within six weeks after rhinectomy [1,22]. Compared to surgery, prostheses offer the great advantage that they can be removed at any time for cleaning and care, but also for inspection and eventual biopsies of the surgical site during oncological follow-up. The other advantages of prostheses are the reduction of overall surgical and hospitalization time, shorter post-operative recovery and the possibility of application at the same time of the resection [1,24]. Besides, almost all patients are potentially suitable candidates for reconstruction with prostheses, including elderly patients with multiple comorbidities, not eligible for surgical reconstruction [22]. The disadvantages include fit and stability of the prosthesis, the possible foreign body sensation, the need for daily cleaning and the formation of crusts. In addition, the prosthesis must be replaced on average every two years due to discoloration, and its cost is often not covered by the healthcare system [12,24]. The main prosthetic complications that emerged in the literature are prostheses failures, periimplantitis, screw losses and infection [8,10,12,18]. 

### 3.4. Aesthetic Outcome

The main challenge for the surgeon is to achieve oncologic radicality while ensuring good aesthetic outcome and patient satisfaction. All seven articles that analyze the aesthetic outcome report encouraging conclusions [1,3,4,7,10,13,14]. 

Total rhinectomy results in a number of potential adverse effects, which must be taken into account. While the somatic effects are mainly local and the procedure is generally well tolerated, the impact on the patients’ self-image and general psychological well-being is severe [1]. Patients therefore require extensive pre-therapeutic counselling and ongoing support.

Of the seven studies that also investigated the aesthetic aspect, three just stated that a good aesthetic result had been achieved in all patients, without specifying how this result had been achieved [4,7,14].

Korfage et al. [10] rated the overall patient satisfaction on a scale of 1 to 10, finding high satisfaction among patients treated with implant-retained nasal prostheses.

A scale of 1 to 10 was also used in the work of Ribuffo et al. [13]; in this case, the assessment was made by both the patient and a plastic surgeon who did not participate in the surgery. High scores were achieved for both two-step and three-step surgery.

D’heygere et al. [1] used the FACE-Q questionnaire [34] to assess the psychosocial impact on patients of the procedure and reconstruction by epithesis. This questionnaire measures three domains: facial appearance, health-related quality of life and adverse effects. The three worst adverse effects were altered sensitivity on touch, numbness of some facial areas and the presence of visible scars. Aesthetically and psychologically, the patients were generally satisfied with the appearance of their nasal epithets and the result of the procedure.

Differently, Becker et al. [3] used the University of Washington Quality of Life questionnaire (UWQOL) [35] and the Nasal Appearance and Function Evaluation Questionnaire (NAFEQ) [36], which is a valid and reliable method for assessing outcomes in patients undergoing nasal reconstruction surgery. The authors highlighted positive results in the areas of overall function, appearance and fit. One of the greatest challenges in nasal prosthesis treatment is ensuring sufficient stability during daily activities. This study showed that a bone-anchored or implant-retained facial prosthesis usually offers good stability and results in a higher satisfaction rate than an adhesive-retained prosthesis.

Therefore, our review reveals that there is no standardized and validated questionnaire for assessing the aesthetic and functional results of patients undergoing nasal reconstruction after oncological rhinectomy.

In addition, it should be emphasized that most of the patients who underwent aesthetic evaluation by questionnaire received reconstruction by prosthesis, and only one paper analyzed the aesthetic result following the FF [13].

This discrepancy makes the comparison of the aesthetic result between the two rehabilitation options very compromised.

According to the authors, good aesthetic quality and patient satisfaction is achieved in the majority of cases. Both prostheses and surgery therefore represent two valid solutions with similar results in this field. Certainly, the lack of a standard method of evaluation, and therefore the use of different assessment parameters by the different authors, makes it very difficult to compare the results obtained in different papers.

## 4. Conclusions

In conclusion, this review shows that both prosthetic and surgical reconstruction are very suitable solutions in terms of surgical and aesthetic outcome for the patient undergoing rhinectomy. On the one hand, surgery probably offers a better benefit in terms of improved skin color and texture match, but it is not applicable to all patients and requires a high level of surgical experience. On the other hand, nasal prostheses allow better oncological surveillance and offer a temporary or permanent alternative to surgery but require daily maintenance and the costs are often not covered by the healthcare system. We therefore believe that both solutions should be proposed to the patient, explaining the advantages and disadvantages of each one well, and then tailoring the reconstructive therapy, choosing the most suitable solution together.

## Figures and Tables

**Table 1:** Literature review.

Author	No.	Sex (M/F)	Average Age (Range) yrs	Histology	Type of Reconstruction	Aesthetic Evaluation	Follow-Up	Complication
Livaoğlu (2009) [2]	6	M: 5 F: 1	(54–85)	BCC 4 OT 2	ALTFF	N	X	X
Ethunandan (2010) [18]	34	M: 24 F: 10	67 (46–86)	BCC 16 SCC 15 OT 3	P	N	31 (4–108) mo	Failure 12
Quetz (2011) [19]	9	X	57 (38–79)	SCC 7 BCC 2	FF	N	24 mo	Venous congestion 2 Cartilage infection 1
Chipp (2011) [4]	14	M: 10 F: 4	64.7 (37–89)	SCC 6 BCC 5 MM 2 OT 1	P 11 RFFF 2 FF 1	Y	30.1 (0–96) mo	none
Paddack (2012) [16]	107	M: 48 F: 59	65.5 (23–85)	BCC 84 SCC 16 OT 7	FF/ NLF	N	X	Flap failure 6 Nasal obstruction 7 Bulky flap surgery 12
Ribuffo (2012) [13]	31	M: 13 F: 18	68 (37–87)	BCC 23 SCC 5 MM 3	FF	Y	>12 mo	Flap necrosis 2 Dehiscence 1
Agostini (2013) [15]	7	M: 5 F: 2	63.4 (58–86)	SCC 4 BCC 3	Bi pedicle FF	N	> 18 mo	none
Seth (2013) [14]	5	M: 2 F:3	64 (54–76)	BCC 2 MM 2 SCC 1	ALTFF	Y	X	X
Lünenbürger (2015) [12]	51	M: 33 F: 18	53 (29–92)	SCC 32 BBC 10 MM 2 OT 7	P	N	>12 mo	Infection 1 Periimplantitis 4
Korfage (2015) [10]	28	M:18 F: 10	68	SCC 20 BCC 2 MM 3 OT 3	P	Y	35.1 mo	Implant failure 2
Subramaniam (2015) [20]	9	M: 3 F: 6	69 (60–78)	SCC 8 OT 1	P: 7 ALTFF: 1 FF: 1	N	5 (0.2–8) yrs	none
Papaspyrou (2016) [8]	22	M: 15 F: 7	65.1 (51–81)	SCC 16 BCC 4 OT 2	P	N	> 24 mo	Revision for screw losses 2
Becker (2017) [3]	43	M: 27 F: 16	61 (37–87)	X	P	Y	45.3 (6–163) mo	none
Girardi (2019) [6]	10	M: 2 F: 8	71.6 (56–87)	SCC 7 BCC 3	FF: 9 P: 1	N	45.7 (18–66) mo	none
Saleh (2020) [17]	7	M: 1 F: 6	46.5 (17–93)	SCC 4 BCC 1 MM 1 OT 1	FF	N	2.46 yrs	Venous congestion 1
Krakowczyk (2020) [7]	48	X	X	BCC 26 SCC 19 MM 1 OT 2	RFFF/ AF	Y	X	Venostasis required surgery 8 Necrosis 4 Partial necrosis 6
D’heygere (2021) [1]	16	M: 9 F: 7	64.1 (49–92)	SCC 14 BCC 2	P	Y	18.3 (4–44) mo	none
Tot	447	M: 215 F: 175	(44.5–71.6)	BCC 177 SCC 174 OT 29 MM 14	Protheses 213 Local Flap 172 Free Flap 62	Y: 7 N: 10		

Abbreviation legend. M: male; F: female; yrs: years; mo: months; Y: yes; N: no; BCC: basal cell carcinoma, SCC squamous cell carcinoma, MM malignant melanoma, OT: other; P: protheses; ALTFF: anterolateral thigh free flap; RFFF: radial forearm free flap; FF: forehead flap; NLF: nasolabial flap; AF: auricular flap; X: missing data.

## Data Availability

Materials are available from the corresponding author upon request.
